# Regulatory B cells are reduced in the blood in patients with granulomatosis with polyangiitis, and fail to regulate T-cell IFN-γ production

**DOI:** 10.1093/cei/uxad021

**Published:** 2023-02-08

**Authors:** Daniel Appelgren, Srinivasulu Puli, Thomas Hellmark, Pierre Pochard, Jacques-Olivier Pers, Jan Ernerudh, Per Eriksson, Mårten Segelmark

**Affiliations:** Department of Health, Medicine and Caring Sciences, Linköping University, Linköping, Sweden; Department of Clinical Sciences in Lund, Nephrology, Lund University and Skåne University Hospital, Lund, Sweden; Department of Clinical Sciences in Lund, Nephrology, Lund University and Skåne University Hospital, Lund, Sweden; LBAI, UMR1227, Univ Brest, Inserm, CHU de Brest, Brest, France; LBAI, UMR1227, Univ Brest, Inserm, Brest, France; Department of Biomedical and Clinical Sciences, Linköping University, Linköping, Sweden; Department of Biomedical and Clinical Sciences, Linköping University, Linköping, Sweden; Department of Rheumatology, Linköping University, Linköping, Sweden; Department of Health, Medicine and Caring Sciences, Linköping University, Linköping, Sweden; Department of Clinical Sciences in Lund, Nephrology, Lund University and Skåne University Hospital, Lund, Sweden; Department of Nephrology, Linköping University, Linköping, Sweden

**Keywords:** regulatory B-cells, autoimmunity, granulomatosis with polyangiitis (GPA), immune regulation, T-cells

## Abstract

Regulatory B (Breg) cells can dampen inflammation, autoreactivity, and transplant rejection. We investigated the frequencies, phenotypes, and function of Breg cells in granulomatosis with polyangiitis (GPA) to gain further knowledge as to whether there are numerical alterations or limitations of their ability to regulate T-cell function. Frequencies and phenotypes of CD24^hi^CD27^+^ and CD24^hi^CD38^hi^ B-cells in the blood were determined with flow cytometry in 37 GPA patients (22 in remission and 15 with active disease) and 31 healthy controls (HC). A co-culture model was used to study the capacity of Breg cells to regulate T-cell activation and proliferation in cells from 10 GPA patients in remission and 12 HC. T-cell cytokine production *in vitro* and levels in plasma were determined with enzyme-linked immunosorbent assay. Frequencies of CD24^hi^CD27^+^ B-cells were reduced both during active disease and remission compared with HC (*P* = 0.005 and *P* = 0.010, respectively), whereas CD24^hi^CD38^hi^ B-cells did not differ. Patient CD24^hi^CD27^+^ B-cells exhibited decreased expression of CD25 but increased expression of PD-L1 and PD-L2 during remission. B-cells from GPA patients regulated T-cell proliferation but failed to regulate interferon (IFN)-γ production (median T-cells alone 222 ng/ml vs. T-cells + B-cells 207 ng/ml, *P* = 0.426). IFN-γ was also elevated in patient plasma samples (*P* = 0.016). In conclusion, GPA patients exhibit altered numbers and phenotypes of CD24^hi^CD27^+^ B-cells. This is accompanied by a disability to control T-cell production of Th1-type cytokines during remission, which might be of fundamental importance for the granulomatous inflammation that characterizes the chronic phase of this disease.

## Teaser

The main findings in the present study are (i) a reduced frequency and (ii) altered phenotype of CD24hiCD27+ putative Breg cells in patients with GPA/PR3-ANCA+ vasculitis compared with HC, and (iii) that induced Breg cells during remission could regulate T-cell proliferation and production of certain cytokines but failed to regulate IFN-? and TNF production. In line with the latter observation, IFN-?, and its induced chemokine CXCL10, were elevated in the circulation.

## Introduction

Granulomatosis with polyangiitis (GPA; previously known as Wegener’s granulomatosis) is a systemic inflammatory disease characterized by necrotizing granulomatosis, small vessel vasculitis, and the presence of antineutrophil cytoplasmic autoantibodies (ANCA) [1]. Nose, lungs, and kidneys are the most commonly affected organs. In GPA, ANCA are mainly directed against proteinase 3 (PR3), and these antibodies can activate neutrophils, which are the most important effector cells in GPA. PR3-ANCA are high affinity and class-switched antibodies requiring cognate interaction between B and T-cells to be produced [2]. Besides promoting autoantibody production, T-cells are abundant in granulomas as well as in affected kidney tissues [3–5]. The fraction of circulating CD4^+^ effector memory T-cells is expanded and seems constantly activated, as mirrored by increased circulating levels of T-cell molecules such as the IL-2 receptor (CD25) and CD30 [6–8]. When GPA develops from localized granulomatosis to generalized vasculitis there is a shift from Th1 polarization toward Th2 and Th17 responses [9–11]. There are also several studies indicating numerical and/or functional abnormalities regarding regulatory T (Treg) cells, or T-cells not responding properly to signals from Treg cells [12–14].

Regulatory B (Breg) cells are subpopulations of B-cells that similar to Treg cells can dampen inflammation, autoreactivity, and transplant rejection [15, 16]. Breg cells operate by regulating T-cell proliferation, skewing T-cell differentiation, inducing Treg cells, and by inhibition of proinflammatory cytokine production by various immune cells [17, 18]. Breg cell function has been strongly associated with production of interleukin (IL)-10, but studies also support regulatory mechanisms through the expression of programmed death ligand 1 (PD-L1) and production of granzyme, IL-35, TGF-β, and Indoleamine 2,3-dioxygenase (IDO) [19–22]. The capacity of Breg cells to regulate immunological responses *in vivo* has primarily been demonstrated in murine models of infectious diseases, tumors, and autoimmune diseases, such as rheumatoid arthritis (RA), systemic lupus erythematosus (SLE), and multiple sclerosis (MS) [17, 18]. It appears that human Breg cells can be found within the pool of innate-like, transitional, and memory B-cells, as well as among plasma cells [16, 23–25]. Breg cells are also associated with expression of induced molecules such as CD25, CD86, TIM-1, and CD9 [26–28]. Although CD11b^+^ B1 B-cells produce IL-10 spontaneously [23], B-cells generally must be stimulated *in vitro* through for example toll-like receptors (TLRs) and/or co-stimulatory molecules (CD40, CD80, CD86) to acquire regulatory features [18, 29]. Both functional and numerical Breg cell defects have been connected to human autoimmune diseases. For instance, CD24^hi^ CD38^hi^ (transitional) Breg cells are functionally defective in SLE, showing impaired secretion of IL-10 and failure to suppress Th1 cells [25]. Furthermore, CD24^hi^ CD27^+^ (memory) Breg cells in patients with bullous pemphigoid are unable to inhibit the release of anti-BP180 autoantibodies, T-cell proliferation, and proinflammatory cytokine production [30].

Measurement of Breg cell subsets in the circulation of AAV patients shows that they are often, but not consistently, reduced during active disease [31–33]. Functional studies have mostly focused on the capacity of B-cells to produce IL-10 upon stimulation *in vitro*, and results are conflicting in relation to disease activity and to healthy controls (HC). IL-10^+^ B-cells have been reported to be reduced during both active disease and remission [34] or only during active disease [35], but there also reports of similar levels compared to HC both during active disease and in remission [36, 37]. Studies on other Breg cell mechanisms than IL-10 production suggest that B-cells from AAV patients behave normally in their ability to regulate T-cell proliferation [38], T-cell production of IFN-γ, and TNF [37] and TNF production by monocytes [36]. Patients with active disease were, however, only included when studying regulation of T-cell proliferation.

To elucidate the role of Breg cells in GPA/PR3-ANCA^+^ vasculitis, an aim of the present study was to investigate the frequencies of Breg cells in the circulation of patients during active disease and remission. Because of the possible heterogeneity in Breg cell definitions, we included phenotypic characterization of the proposed CD24^high^CD27^+^ Breg cell population. An additional aim was to assess the ability of induced Breg cells during remission to regulate T-cell proliferation and proinflammatory cytokine production in a B-cell/T-cell co-culture system.

## Material and methods

### Study population

Patient and healthy controls (HC) study populations were recruited from the Departments of Rheumatology and Nephrology at the University Hospital of Linköping, Sweden. A total of 37 GPA patients and 31 HC were included in the phenotype study (Table 1), no patient nor HC was sampled twice. For the functional studies, 10 GPA patients and 12 HC were included, and all these patients were in remission with a Birmingham vasculitis activity score (BVAS) of 0 (Table 2). Data on disease activity, disease extension, treatment, and ANCA serology for both cohorts were obtained from medical records. Informed consent was obtained from all subjects in accordance with the declaration of Helsinki, and the study was approved by the Regional Ethical Review Board in Linköping.

**Table 1:** Study population for B-cell phenotyping

	Healthy control	GPA remission	GPA active	*P*-value
Number of individuals: *n* (female/male)	31 (17/14)	22 (11/11)	15 (7/8)	0.861
Age: median years (range)	60 (26–85)	69 (22–87)	65 (29–93)	0.369
ANCA at diagnosis: PR3	na	22	15	
Disease onset/relapse	na	na	8/7	
Organ involvement				
ENT, % (*n*) [ever/sample]	na	86 (19) [19/na]	73 (11) [11/11]	0.408
Lungs, % (*n*) [ever/sample]	na	55 (12) [12/na]	73 (11) [11/7]	0.314
Kidneys, % (*n*) [ever/sample]	na	45 (10) [10/na]	53 (8) [8/5]	0.743
Nervous system, % (*n*) [ever/sample]	na	23 (5) [5/na]	33 (5) [5/2]	0.708
Treatment at time of sampling				
Prednisolone, % (*n*)	na	59 (13)	60 (9)	1.000
Prednisolone, median mg/day (range)	na	2.5 (0-5)	2.5 (0–30)	0.230
Azathioprine, % (*n*)	na	23 (5)	0 (0)	0.067
Methotrexate, % (*n*)	na	32 (7)	3 (2)	0.262
Mycophenolate mofetil % (*n*)	na	9 (2)	0 (0)	0.505
No immunosuppressive therapy, % (*n*)	na	14 (3)	40 (6)	0.118
Prior treatment: Cyclophosphamide: % (*n*)	na	82 (18)	33 (5)	0.005
Rituximab: % (*n*)	na	41 (9)	27 (4)	0.491
Time since rituximab at sampling in treated patients: median months (range)	na	26 (12–65)	15 (11–94)	0.434
Neither cyclophosphamide or rituximab: % (n)	na	9 (2)	60 (9)	0.002
BVAS: median (range)	na	0	14 (2–26)	

AAV: ANCA-associated vasculitis; MPO: myeloperoxidase; MPA: microscopic polyangiitis; ENT: ear, nose, and throat; na: not applicable. The Chi-square test was used to compare number of female/male whereas Fisher´s exact test was used to compare other discrete variables between the groups. The Kruskal–Wallis test was used to compare age, whereas Mann–Whitney *U* test was used to compare other continuous variables between the groups. Significance assumed at *P* < 0.05.

**Table 2:** Study population for B-cell functional study

	Healthy controls	GPA remission	*P*-value
Number of individuals: *n* (female/male)	12 (8/4)	10 (7/3)	1.000
Age: median years (range)	69 (37–73)	59 (27–77)	0.096
ANCA at diagnosis: PR3/MPO	na	10/0	—
Lymphocytes: median × 10^9^/L (range)	2 (0.8–3.4)	1.7 (0.9–3.2)	0.445
Creatinine: median µmol/L (range)	76 (59–137)	86.5 (62–208)	0.244
C-reactive protein: median mg/L (range)	<5	<5 (<5–12)	—
Treatment at time of sampling			
Prednisolone, % (*n*)	na	40 (4)	—
Prednisolone, median mg/day (range)	na	5 (3.75–5)	—
Methotrexate, % (*n*)	na	60 (6)	—
No immunosuppressive therapy, % (*n*)	na	10 (1)	—
Prior treatment			
Cyclophosphamide: % (*n*) Rituximab: % (*n*)	na na	60 (6) 70 (7)	— —
Time since rituximab at sampling in treated patients: median months (range)	na	70 (59–139)	—

AAV: ANCA-associated vasculitis; MPO: myeloperoxidase; MPA: microscopic polyangiitis; na: not applicable. Fisher’s exact test was used to compare discrete variables and Mann–Whitney *U* test for continuous variables between the groups. Significance assumed at *P* < 0.05.

### Cell isolation

White blood cell counts (WBC) and absolute numbers of circulating lymphocytes were analysed at the Clinical Chemistry Laboratory at the Linköping University Hospital with a Cell-Dyn Sapphire (Abbot, Abbot Park, IL, USA). Peripheral blood mononuclear cells (PBMC) were isolated from heparinized blood by Ficol (GE Healthcare, Uppsala, Sweden) gradient centrifugation. PBMC used for the phenotype analysis were resuspended in medium containing 90% fetal bovine serum (FBS, EuroBio Scientific, Les Ulis, France) and 10% dimethyl sulfoxide (Sigma-Aldrich, St Louis, MO, USA) and put in a Mr Frosty Freezing container (Thermo Fisher Scientific, Waltham, MA USA), and stored in a −70°C freezer for 24–48 h. Samples were then transferred for cryopreservation in liquid nitrogen to be used later for flow cytometry analysis. For functional studies, T and B-cells were isolated from fresh PBMC isolated as above. Total T and B-cells were enriched from PBMC through negative selection using the EasySep Human B and T-cell Isolation Kits from Stemcell Technologies, respectively, according to the manufacturer’s instructions (Stemcell Technologies, Saint Egreve, France). Purity of isolated T and B-cells was analysed with flow cytometry using a Gallios Flow Cytometer (Beckman coulter, Brea, CA USA). B-cell (CD19^+^) and T-cell (CD3^+^) purity were constantly high in samples from both patients and HC; median purity exceeded 99% for both T-cells (range 96.5–99.8%) and B-cells (range 97.9–100%).

### Flow cytometry to assess frequency and phenotype of Breg cells in the circulation

PBMC were thawed in a 37°C water bath before being washed twice in RPMI 1640 (Gibco Paisley, UK) containing 10% FBS (EuroBio). Isolated B-cells were resuspended in a staining buffer (PBS with 0.1% FBS) before labelling of surface molecules to compare the frequencies of two different proposed Breg cell populations; CD24^hi^CD27^+^ and CD24^hi^CD38^hi^ B-cells. Additionally, CD24^hi^CD27^+^ B-cells were characterized by analysing surface molecule expression of activation markers (CD25 and CD38), co-inhibitory molecules (PDL1 and PDL2), and the co-stimulatory molecule CD86. Gating strategies are presented in Supplementary Figs. S1 and S2. Fluorochrome minus one (FMO)-controls were used to set suitable gates to determine positivity for a particular surface molecule and were repeated occasionally. CD25 was purchased from Becton Dickinson (Franklin Lakes, NJ, USA) whereas the rest of the antibodies were bought from BioLegend (San Diego, CA, USA). Samples were analyzed using a Gallios Flow Cytometer (Beckman Coulter). The freezing and thawing procedure did not affect the proportions of the investigated B-cell subsets (HC, *n* = 4, data not shown). Absolute numbers of B-cell populations were based on the proportion (%) of B-cells within the lymphocyte population combined with the absolute number of lymphocytes from the WBC.

### T- and B-cell co-culture assay to study Breg cell function

To determine the regulatory capacity of peripheral B-cells on T-cell activation we used an assay previously described [20, 29, 39]. Briefly, T-cells were labelled with carboxyfluorescein succinamidyl ester (CFSE, Invitrogen, CA; USA), 5 µM for 5 min, to enable monitoring of proliferating cells by dye dilution. Cells were resuspended in RPMI 1640 containing 10% FBS (EuroBio), 2 mM l-glutamine (Invitro Life Technologies, Noble park north, Australia), 200 U/ml penicillin, and 100 µg/ml streptomycin and cultured in 96-well plates (Eppendorf, Hamburg, Germany) for 96 h. 4 × 10^4^ T-cells were seeded on plates coated with affinipure F(abʹ)2 fragment of goat anti-mouse IgG antibodies (Jackson Immunoresearch, Cambridgeshire; UK), and stimulated with soluble anti-CD3 (OKT3; 0.2 μg/ml; Bio Legend, France) and anti-CD28 (CD28; 0.2 μg/ml; Beckman coulter, California, USA) antibodies. For co-culture experiments, 4 × 10^4^ B-cells were added to the T-cells, with or without CpG-B oligodeoxynucleotides of class B (CpG-B, ODN 2006; 0.25µM; Cayla Invivogen, Toulouse, France). All cell culture setups (T-cells alone or together with B-cells) were run in duplicates or triplicates. T-cell proliferation (total T-cells, CD4 T-cells, and CD8 T-cells) was evaluated with a Gallios Flow Cytometer (Beckman Coulter). Gating strategy is presented in Supplementary Fig. S3. Antibodies were bought from BioLegend (San Diego, CA, USA). T-cell proliferation was measured as proliferation index (PI) and division index (DI), as described in the FlowJo v10 documentation: https://docs.flowjo.com/flowjo/experiment-based-platforms/proliferation/ (16 January 2022, last accessed). Here, PI is defined as the average number of cell divisions in cells that have started to proliferate (total number of divisions/number of cells that went into division), whereas DI is the average number of cell divisions that a cell in the original population has undergone including cells that never started to proliferate (total number of divisions/number of cells at start of culture). When establishing the co-culture assay at our laboratory, we conducted T-cell density control experiments to ensure that B-cells inhibited T-cell proliferation and were not an effect of increased cell density as well as tested that an increased number of B-cells resulted in more inhibition (Supplementary Fig. S4).

### ELISA to measure cytokines in cell culture supernatants and plasma

Commercial kits were used to detect IL-17A, IL-5, IFN-γ, TNF, and IL-10 (BioLegend) in cell culture supernatants, and IFN-y, TNF, and CXCL10 (BioLegend) in plasma samples. All procedures were performed according to the manufacturer instructions. Briefly, 96-well Nunc Maxisorp plates (Bio Legend) were coated over-night at 4°C with antibodies against the cytokines and chemokine of interest. After incubation with standards and sample supernatants, a biotinylated detection antibody was added. Subsequently, avidin-HRP was added followed by TMB substrate solution, producing a blue colour relative to the cytokine concentration. Finally, acid solution (2N H_2_SO_4_) was added to stop the reaction. A VMAX^®^ plate reader (VWR International, Pennsylvania; USA) was used to measure OD values at 450 nm.

### Statistics

D’Agostino and Pearson omnibus normality tests were used to determine whether the data on continues variables had a Gaussian distribution or not. Since all data had a non-Gaussian distribution, the Kruskal–Wallis test followed by Dunn’s multiple comparison tests was used when comparing more than two groups with independent observations, whereas Mann–*Whitney U* test were applied in comparisons between two groups with independent observations. Wilcoxon test was applied to compare paired data. Fisher’s exact test or the Chi-squared test was used to compare discrete variables. For correlation analyses, Spearman’s rank correlation coefficient, *r*_s_, was calculated. Data are presented as median with interquartile range (IQR). A *P* value of <0.05 was considered statistically significant. Data were analyzed using GraphPad Prism, version 6.02 (GraphPad Software, San Diego, CA, USA).

## Results

### Frequencies of circulating Breg cells are reduced in patients with GPA

**Figure 1:**
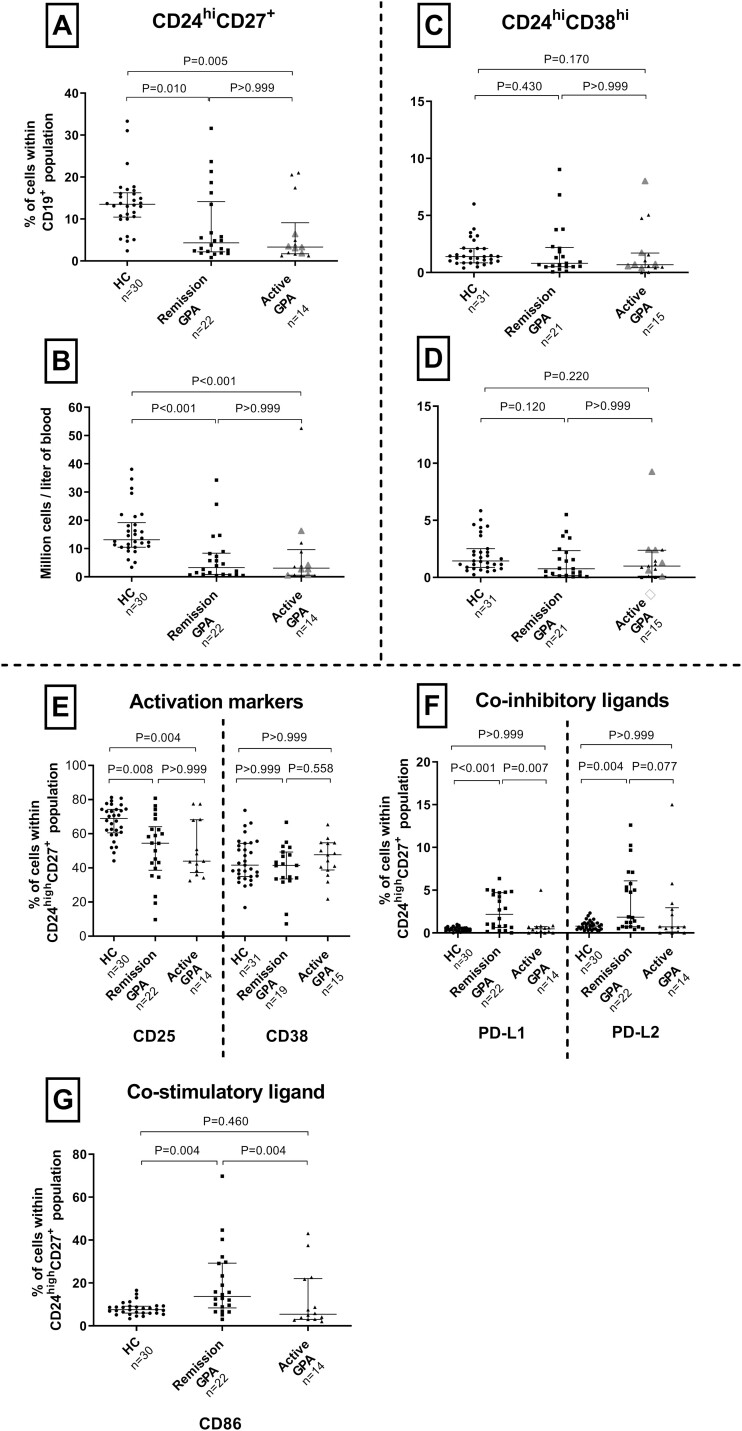
Frequency and phenotype of Breg cells in the circulation. (**A and B**) Both the percentage of CD24^hi^CD27^+^ B-cells within the B-cell population and their absolute numbers were reduced in the circulation of patients irrespective of disease activity compared with HC. (**C and D**) For CD24^hi^CD38^hi^ B-cells, neither the absolute numbers nor their proportions of these differed between the groups. Additional phenotyping was conducted on CD24^hi^CD27^+^ B cells. **(E)** CD25^+^ cells were reduced in GPA patients both during remission and active disease compared with HC, but expression of CD38 did not differ between the groups. **(F and G)** There were more cells expressing the co-inhibitory ligands PD-L1 and L2, and the co-stimulatory ligand CD86, during remission compared with both patients with active disease and HC. Grey triangles indicate six treatment-naïve patients with active disease (i.e. disease debute). Bars indicate median and inter-quartile range. *P < 0.05, **P < 0.01, ***P < 0.001. HC: healthy controls, GPA: granulomatosis with polyangiitis.

To study the frequency of putative Breg cells we recruited 37 GPA patients and 31 HC. All patients were positive for PR3-ANCA at diagnosis (Table 1). Patients with active disease (*n* = 15) were sampled before starting induction therapy with cyclophosphamide or rituximab (RTX), and most of them were without or with only low doses of steroids (Table 1). Among the 22 patients sampled in remission, nine had previously received RTX, with a median time from treatment to sampling of 26 months, and 18 had received cyclophosphamide (Table 1). The putative Breg cells within the memory B-cell pool, identified as CD24^hi^CD27^+^, were decreased in GPA patients irrespective of disease activity compared with HC, both when expressed in absolute numbers or as the percentage within the total B-cell pool (Fig. 1A and B). In contrast, for the transitional B-cells, characterized as CD24^hi^CD38^hi^, neither the absolute numbers nor the proportion of these cells within the B-cell population differed significantly between GPA patients and HC (Fig. 1C and D). There were no differences for any of the B-cell subpopulations, neither in absolute nor in relative terms, between patients with active vasculitis and those in stable remission (Fig. 1A–D).

### Altered phenotype of Breg cells in GPA patients depends on disease activity

To further characterize CD24^hi^CD27^+^ B-cells, i.e. Breg cells in the memory B-cell pool, we analyzed the expression of surface molecules related to activation, co-stimulation, and co-inhibition. CD25 is part of the IL-2 receptor and constitutes an activation marker. Furthermore, CD25^+^CD27^+^ B-cells have been suggested to constitute a Breg cell population. We observed CD25 to be highly expressed on CD24^hi^CD27^+^ B-cells from HC, and this marker was reduced during both remission and active disease (Fig. 1E). CD38 is a calcium signaling protein, and its expression varies in a biphasic pattern during development. It is also regarded as an activation marker, and both plasmablasts (CD38^+^CD27^+^) and transitional B-cells (CD24^hi^CD38^hi^) produce IL-10. The expression of this molecule within CD24^hi^CD27^+^ B-cells did not differ between patients and HC (Fig. 1E). Very few CD24^hi^CD27^+^ B-cells expressed the co-inhibitory ligands programmed death-ligand 1 and 2 (PD-L1/2) (Fig. 1F) or the co-stimulatory molecule CD86 (Fig. 1G). There was, however, a significant increased expression of CD86, PD-L1, and PD-L2 on CD24^hi^CD27^+^ B-cells during remission compared to HCs and patients with active disease (Fig. 1F and G). Expression of PD-L1 and PD-L2 during remission seemed to generate two distinct groups of patients (high/low). However, the limited sample size and follow-up time does not allow for a meaningful comparison of relapse rates between the two groups.

### B-cells from both healthy controls and from GPA patients regulate T-cell proliferation

**Figure 2:**
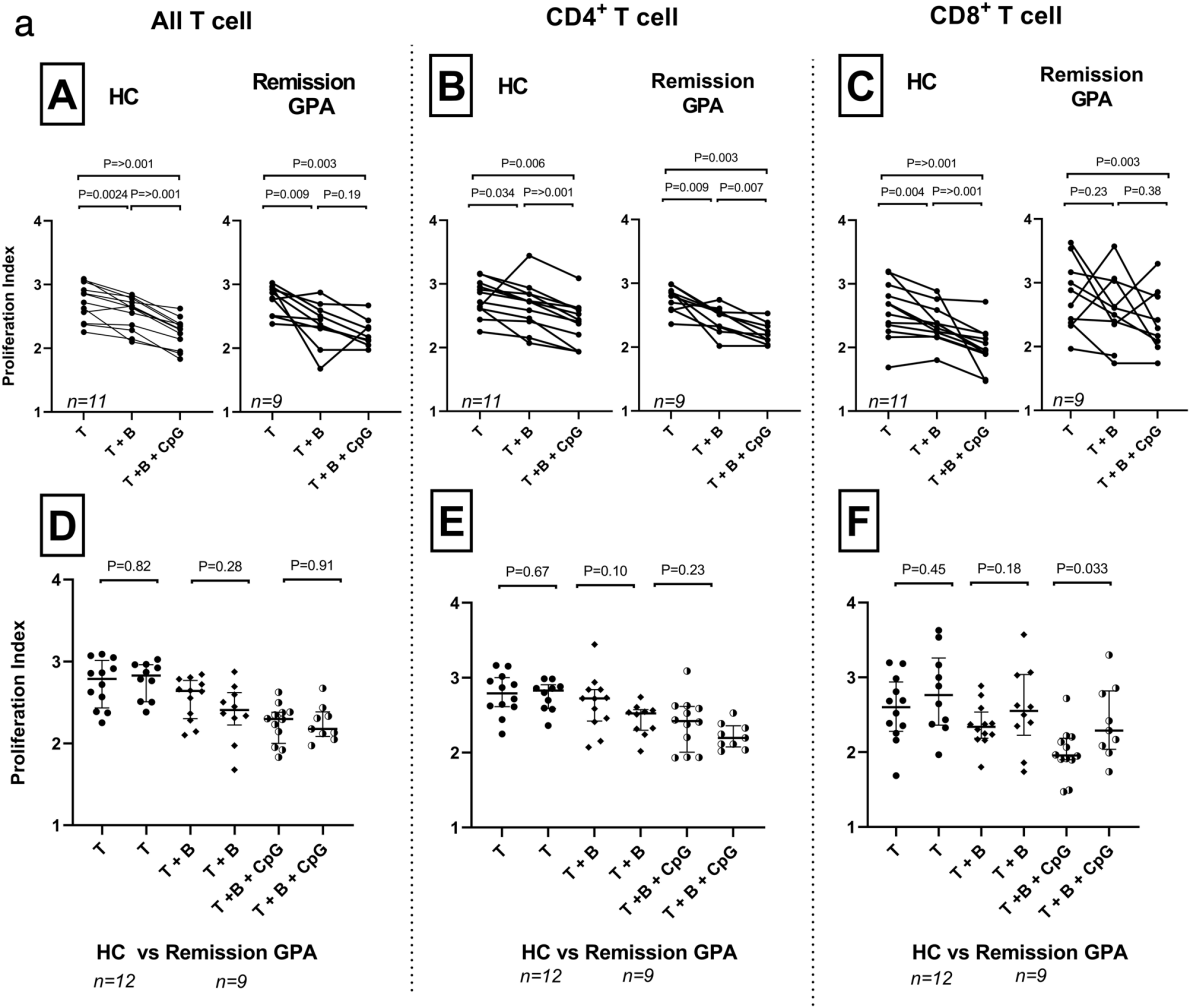

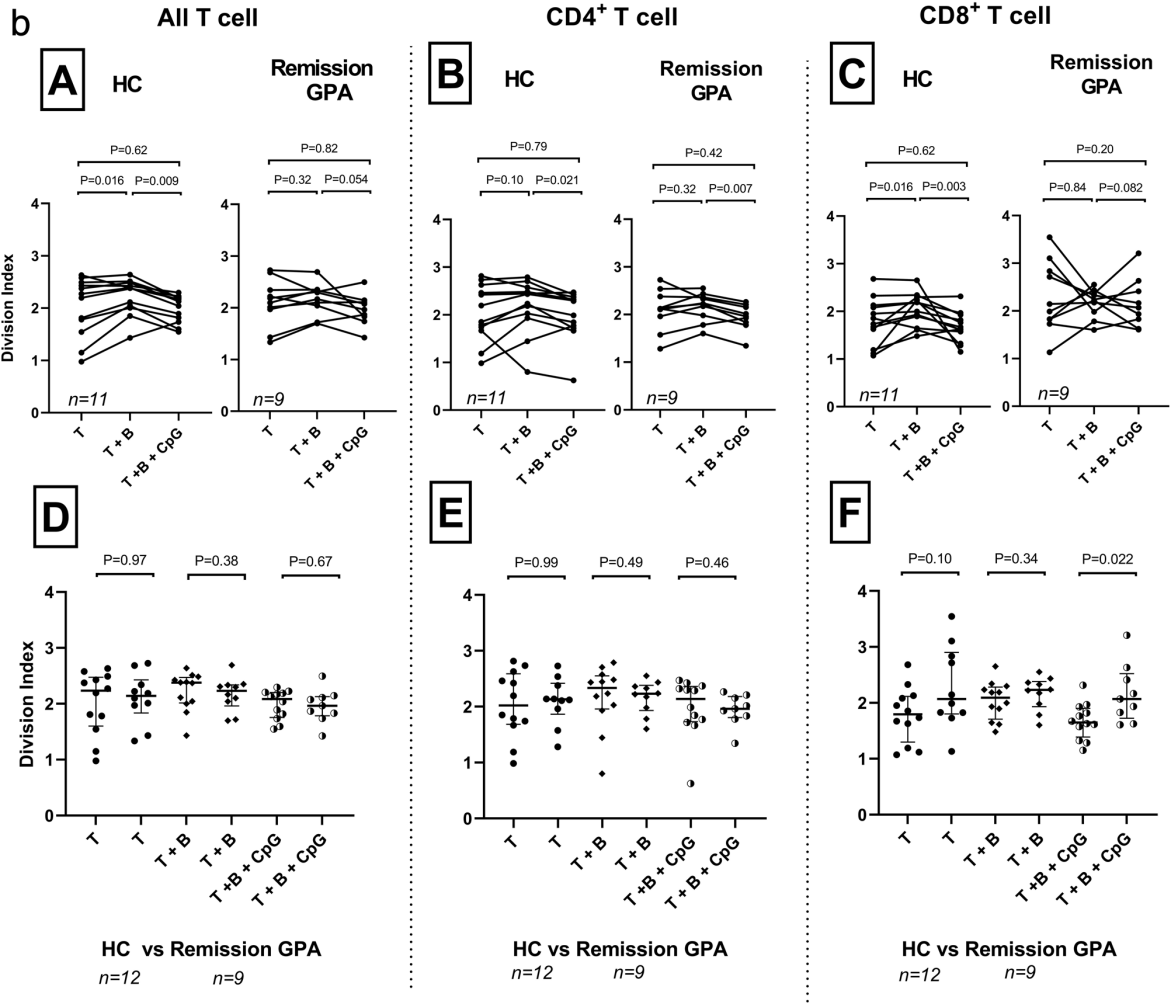
B-cell regulatory effect on T-cell proliferation *in vitro.* PI, the average number of cell divisions in cells that have started to proliferate, is depicted in Fig. 2a and DI, the average number of cell divisions that a cell in the original population has undergone including cells that never started to proliferate, in Fig. 2b. The same data is shown in relation to inhibition of proliferation (A–C) and in comparison between GPA patients and HC (D–F). B-cells in this co-culture model regulate proliferation rate in T-cells that have started to proliferate (PI), rather than preventing them to enter proliferation (DI). PI and DI for CD8^+^ T-cells were lower in cultures with CpG-activated B-cells from HC compared with GPA patients. Bars indicate median and inter-quartile range. *P < 0.05, **P < 0.01, ***P < 0.001. PI; proliferation index, DI; division index, HC; healthy controls, GPA; granulomatosis with polyangiitis

To discern the functional importance of the differences in numbers, proportions, and phenotypes of the Breg cells, we investigated the ability of B-cells from patients and HC to regulate T-cell proliferation and cytokine release. We recruited 12 HC and 10 GPA patients that had been in stable remission for a minimum of 3 years, and as seen in Table 2 this group of patients was younger compared to the phenotype cohort [29]. To study whether such induced Breg cells could inhibit T-cell proliferation we used both proliferation index (PI) and division index (DI) (Fig. 2a and b). PI shows the average number of divisions in T-cells that have started to proliferate, while DI shows the average number of divisions for all cells, including non-dividing cells. PI for the whole T-cell population was decreased by the addition of unstimulated B-cells from HC, and addition of CpG-B stimulated B-cells caused an even more robust inhibition of T-cell proliferation, evident also in both CD4 and CD8 T-cells. PI for CD8^+^ T-cells, but not total T-cells or CD4^+^ T-cells, was lower in cultures with CpG-stimulated B-cells from HC compared with GPA patients (Fig. 2aF). DI revealed that unstimulated, but not CpG-stimulated, B-cells from HC promoted T-cell proliferation, resulting in a higher DI than for T-cells alone (Fig. 2b A–C). For CD8^+^ T-cells DI was lower for CpG-stimulated B-cells from HC compared with cells from GPA patients (Fig. 2bF). The difference between PI and DI is expected in this co-culture system as Breg cells need to be induced and do not prevent T-cells to start proliferating, but do slow the rate of the subsequent proliferation.

### B-cells from patients with GPA fail to regulate T-cell IFN-γ and TNF production

**Figure 3:**
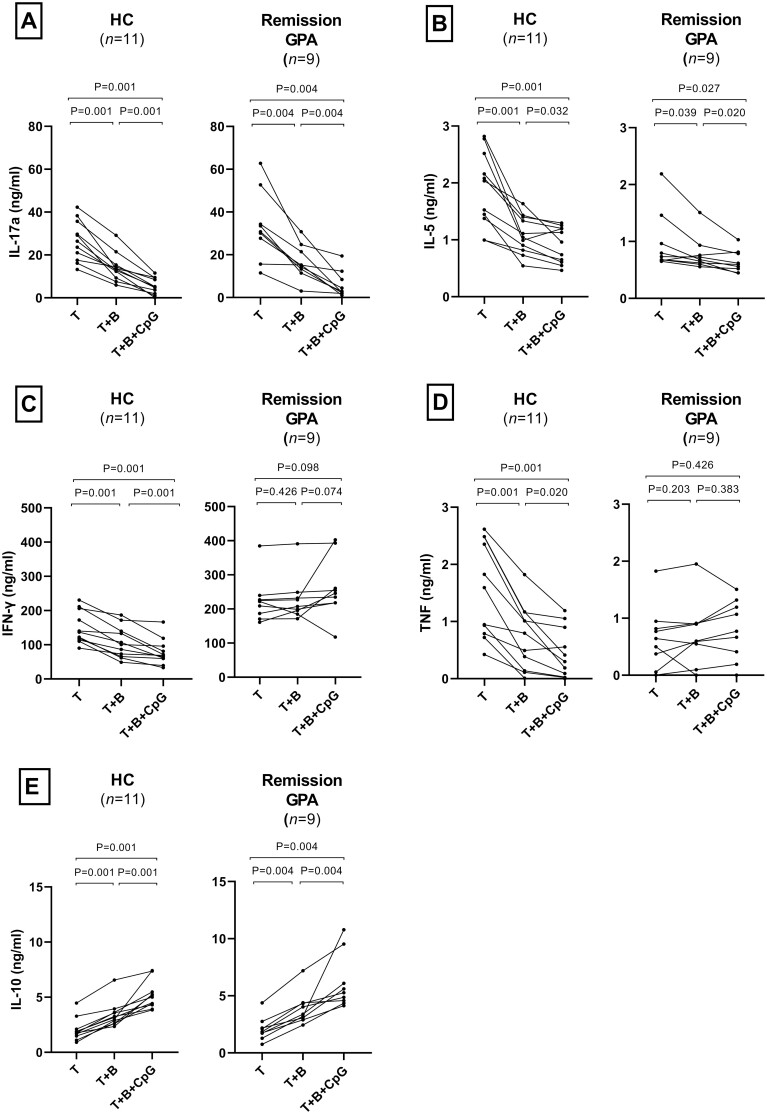
B-cell regulatory effect on T-cell cytokine production *in vitro.* Production of **(A)** IL-17A and **(B)** IL-5 by activated T-cells were reduced in the presence of B-cells, with or without CpG-B, for both HC and GPA patients in remission. Production of **(C)** IFN-γ and **(D)** TNF were, however, only reduced in the presence of B-cells from HC, not patients, also with CpG-B stimulated B-cells. **(E)** IL-10 production was elevated in the supernatants when T-cells were co-cultured with B-cells alone or in the presence of CpG-B, for both patients and HC. Bars indicate median and inter-quartile range. *P < 0.05, **P < 0.01, ***P < 0.001. HC; healthy controls.

We analysed the suppressive capacity of B-cells on T-cell cytokine production during co-culture. IL-17A and IL-5 secretion was reduced when T-cells were co-cultured with either unstimulated or CpG-B stimulated B-cells from both HC and GPA patients (Fig. 3A and B). In contrast, IFN-γ and TNF production were reduced in the presence of B-cells from HC only (Fig. 3C and D). The dysregulation of IFN-γ and TNF production in patients was, however, not paralleled by a difference in IL-10 production compared with HC. IL-10 production was considerably increased for both HC and patients when T-cells were cultured with either unstimulated or CpG-B stimulated B-cells (Fig. 3E).

### Cytokine and chemokine levels in the circulation

**Figure 4:**
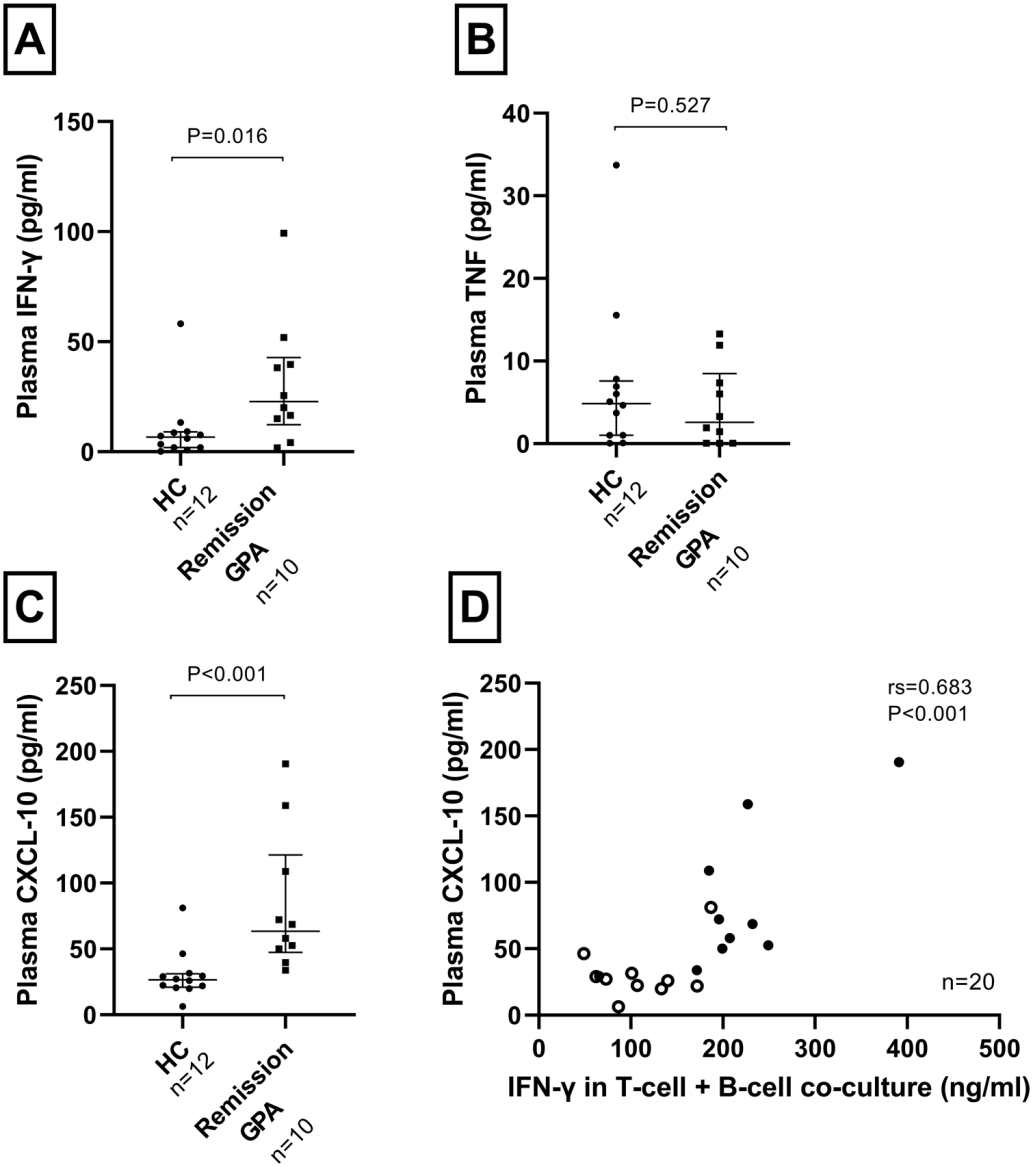
Plasma cytokine and chemokine levels. Samples from GPA patients exhibited significantly higher levels of **(A)** IFN-γ and **(C)** CXCL-10/IP-10 compared to HCs, whereas **(B)** TNF levels did not differ between GPA patients and HC. **(D)** Correlations analysis between IFN-γ released by activated T-cells *in vitro* when co-culture with unstimulated B-cells and CXCL-10 in the blood circulation. Open and closed circles indicate HC and GPA patient samples, respectively. Bars indicate median and inter-quartile range. *P < 0.05, **P < 0.01, ***P < 0.001, HC; healthy controls

To assess if the failure to regulate Th1-related cytokines *in vitro* was mirrored in plasma, we measured the levels of IFN-γ, TNF, and CXCL10. We used plasma obtained at the same time points as the cells used in the functional co-culture experiments. Production of the chemokine CXCL10 is induced by IFN-γ, but with a longer half-life than the cytokine. Notably, increased plasma levels were noted for IFN-γ and CXCL10, but not for TNF compared with HC (Fig. 4A–C). We observed a positive correlation between IFN-γ production by activated T-cells *in vitro* (cultured with unstimulated B-cells) and plasma levels of CXCL10 (*r*_s_ = 0.68; *P* < 0.001) (Fig. 4D).

## Discussion

The main findings in the present study are (i) a reduced frequency and (ii) altered phenotype of CD24^hi^CD27^+^ putative Breg cells in patients with GPA/PR3-ANCA^+^ vasculitis compared with HC, and (iii) that induced Breg cells during remission could regulate T-cell proliferation and production of certain cytokines but failed to regulate IFN-γ and TNF production. In line with the latter observation, IFN-γ, and its induced chemokine CXCL10, were elevated in the circulation.

Our result of reduced levels of CD24^hi^CD27^+^ Breg cells confirms two previous studies [36, 40], which both consisted mainly of GPA patients (100% and 93%, respectively), while another study containing equal numbers of GPA and MPA patients did not observe this difference [35]. It is possible that the reduced frequency of CD24^hi^CD27^+^ B-cells is a specific feature for GPA/PR3-ANCA+ vasculitis, and that lumping different AAV subsets may provide false negative results. We observed no statistical differences in the frequency of CD24^hi^CD38^hi^ B-cells between GPA and HC. Others have also observed similar levels of CD24^hi^CD38^hi^ B-cells in GPA patients during remission and HC [36, 40], but one of the studies showed reduced levels in the circulation during active phase compared to both patients in remission and HC [36]. Reduced proportions of CD24^hi^CD38^hi^ B-cells have, however, been shown in PR3-ANCA^+^ patients both during remission and active disease compared with HC [37], or only among those on maintenance therapy [41]. Another study instead reported an increased proportion of CD24^hi^CD38^hi^ B-cells in PR3-ANCA^+^ patients during remission compared to HC, whereas active patients did not significantly differ from those in remission or HC [35]. They did, however, also measure CD24^hi^CD38^hi^ B-cells expressing CD5 and found them reduced during active disease. Overall, these studies suggest Breg cells found within the memory B-cell pool have an altered distribution in GPA/PR3-ANCA^+^ AAV compared to controls. Differences between studies may apart from differences in disease activity, treatment, and patient characteristics also depend on minor differences in gating strategies and other technical issues.

Within the group of CD24^hi^CD27^+^ Breg cells, we found that GPA/PR3-ANCA^+^ vasculitis patients during both active disease and remission possessed fewer CD25^+^ cells. Altered expression of CD25 could be relevant in this disease as CD25^+^ B-cells are connected to various immune regulatory mechanisms, such as production of IL-10 and TGF-β, and cell-contact dependent regulatory mechanisms [27, 42, 43]. A study showing that IL-10-producing B-cells are enriched within the CD24^hi^CD27^+^ population, also showed that these cells express CD25 to a larger extent than cells not producing IL-10 (24). Studies in AAV have shown contradicting data on levels of IL-10-producing B-cells but this may be explained by differences in disease activity [34–37]. CD80 and CD86 constitute co-stimulatory molecules involved in T-cell activation but blocking them can also reduce the potential for B-cell regulation [25, 29]. Other molecules involved in immune regulation include the inhibitory ligands PD-L1 and PD-L2 [44], and interestingly we observe an increased expression of CD86, PD-L1, and PD-L2 on CD24^hi^CD27^+^ B-cells during remission. An increased B-cell expression of CD86 during remission confirms a previous study from our laboratory [31]. Others have shown that PD-1, the receptor for PD-L1 and PD-L2, PD-1, is upregulated on CD4^+^ T-cells during remission in GPA, but that T-cells at vasculitic lesions lack PD-1 expression [14]. Measurements of certain Breg cell populations in the circulation, or their phenotypes, could potentially be valuable as biomarkers for the risk of relapse. However, this remains to be determined.

We observed that B-cells from PR3-ANCA^+^ GPA patients could regulate T-cell proliferation and production of IL-17A and IL-5, but not IFN-γ and TNF, compared with HC. It has previously been shown for this co-culture model, through blocking and trans-well experiments, that Breg cells are induced following interaction with T-cells and that T-cell proliferation and production of IFN-γ and TNF are differentially regulated. B-cell production of TGF-β and indoelamine 2,3-dioxygenase are critical to regulate T-cell proliferation, and include induction of Treg cells, whereas IL-10 regulates IFN-γ and TNF production [20, 29]. As IFN-γ and TNF were not regulated by patient B-cells in our study, it is interesting to note that the levels of IL-10 were similar in the co-culture supernatants with cells from patients and HC. We did not investigate the kinetics of IL-10 production during culture and to what extent that could affect proinflammatory cytokine production. Even though it has been shown that blockade of IL-10 alone in this co-culture model revert production of IFN-γ and TNF [20, 29], we cannot rule out that other cytokines, not measured in our experiments, may have contributed to the inability to inhibit IL-10 production. A third explanation for our results could also be that the GPA T-cells did not respond properly to the produced IL-10. Such a mechanism, with resistance to IL-10-mediated inhibition of IFN-γ production, has previously been described in patients with active rheumatoid arthritis (RA). The dysregulated production of IFN-γ *in vitro* for GPA patients was also reflected in plasma samples from these patients, with increased levels of IFN-γ and its induced chemokine CXCL10 compared with HC. IFN-γ is a proinflammatory cytokine that drives inflammation through various mechanisms, such as by promoting Th1 and follicular T helper cell responses, M1 macrophage polarization, and B-cell antibody-class-switch and production [38, 43, 45], and contributes to granuloma formation [3]

Todd *et al.* have previously investigated the effect of B-cells on T-cell IFN-γ and TNF production in two GPA and three MPA patients [37]. In the study, they also measured IL-10 production *in vitro*, and in line with our results B-cells from patients were found to produce IL-10 to a similar extent as HC [37]. However, in contrast to our results Breg cells were found to regulate T-cell production of IFN-γ and TNF to a similar extent in patients and HC. The studies differ in several aspects besides the difference in patient selection. They cultured isolated CD24^hi^CD38^hi^ B-cells, naïve B-cells, or memory B-cells with CD4^+^CD25^−−^ T-cells. In our study, we used all B- and T-cells in the co-culture, and because of this difference we do not know the contribution of the various B-cell subsets. In line with previous reports, Todd *et al.* report that both naïve and memory B-cells promote T-cell IFN-γ and TNF production [37]. The mechanism for this was not described, but B-cells can produce a variety of cytokines to do so, such as IL-12, IFN-γ, and TNF [46, 47]. Thus, B-cells could contribute to the levels of IFN-γ and TNF in the co-culture supernatants in our study both as producers of these and via activation of T-cells. Previous reports on GPA have, however, shown patient B-cells to do so only to a similar, or lower, extent as B-cells from HC [37, 40].

Limitations of our study include that we did not phenotype all possible Breg subpopulations in the circulation, and we did not assess the capacity of those we measured to produce IL-10. On the other hand, we focused on two of the most well-studied phenotypes. Another limitation is that we did only co-culture T-cells with the total pool of B-cells, whereas culture also with various subpopulations of B-cells could have provided more information on the dysfunctional regulation of patient T-cell activation. It is difficult to draw conclusions on B-cell intrinsic regulatory function when studying them all since subpopulations, according to our and other studies, can differ in numbers during disease. By including all B-cells, however, we assess the total Breg cell activity, which is relevant for the *in vivo* situation. We chose to study Breg cells induced by activated T-cells, with or without addition of CpG, as these are known stimuli for this purpose [18, 29], and that we consider these interactions relevant during GPA. There are, however, also other stimuli in different combinations that can induce Breg cells [17, 18]. Criss–cross experiments where patient B-cells are cultured with T-cells from HC, and vice versa, would have been useful to discern whether patient T-cells are less sensitive to IL-10, as discussed above, and intracellular staining of cytokines to obtain information on what cells that did, and did not, produce the specific cytokines of interest. These aspects are both limitations of our study. B-cells and T-cells primarily most probably interact in secondary or tertiary lymphoid organs where the important regulation of T-cells occurs; both regarding T-cell differentiation into various subsets and the effect of follicular T-helper cells on B-cell differentiation into plasma and memory B-cells. It is therefore an important limitation of our study that we only use cells from peripheral blood. Strengths of our study, however, include focusing on PR3-ANCA^+^ GPA patients with little or no immunosuppression, and in our phenotype study, all patients with active disease were sampled before high-dose corticosteroid and cytotoxic treatment was started.

In conclusion, we found that GPA/PR3-ANCA^+^ patients have altered numbers and phenotypes of putative Breg cell subpopulations. During remission, this is accompanied by a disability to control T-cell production of Th1-type cytokines, which might be of fundamental importance for granulomatous inflammation that characterizes the chronic phase of this disease. Future research will reveal if this cytokine production can become a pharmacological target to prevent systemic flares.

## Supplementary Material

uxad021_suppl_Supplementary_Material

## Data Availability

The data underlying this article will be shared on reasonable request to the corresponding author.

## Abbreviations

ANCA: anti-neutrophil cytoplasmic autoantibody
Breg cell: regulatory B-cell
BVAS: Birmingham vasculitis activity score
CFSE: carboxyfluorescein succinamidyl ester
DI: division index
FBS: fetal bovine serum
GPA: granulomatosis with polyangiitis
HC: healthy control
IDO: indoleamine 2,3-dioxygenase
IL: interleukin
IQR: interquartile range
MS: multiple sclerosis
PBMC: peripheral blood mononuclear cells
PD-L1: programmed death ligand 1
PI: proliferation index
PR3: proteinase 3
RA: rheumatoid arthritis
RTX: rituximab
SLE: systemic lupus erythematosus
TLRs: toll-like receptors
Treg cell: regulatory T-cell
WBC: white blood cell count
