# Supplementary material for: Regulatory B cells are reduced in the blood in patients with granulomatosis with polyangiitis, and fail to regulate T-cell IFN-γ production
Source: Clin Exp Immunol. 2023 Feb 8;213(2):190–201. doi: 10.1093/cei/uxad021 (PMC10361745; doi:10.1093/cei/uxad021)
Supplement: uxad021_suppl_Supplementary_Material [file uxad021_suppl_supplementary_material.docx]

## Fig S1

CD24^hi^CD27^+^

B-cells

B-cells

CD24^hi^CD38^hi^

3.32%

10%

CD24

CD24

CD38

CD27

**** CD27 FMO control

CD24^hi^CD27^+^

0.23%

CD24

CD27

**Fig S1. Flow cytometry gating strategies for CD24^hi^CD27^+^ and CD24^hi^CD38^hi^ B-cells to determine their frequency in the blood.** Doublet discrimination was conducted on PBMC before gating the lymphocyte population. Within the lymphocyte population, B cells were defined as CD19^+^ and selected for further analysis of CD24^hi^CD27^+^ and CD24^hi^CD38^hi^ B cells. FMO; fluorochrome minus one.

## Fig S2

45%

CD25

11%

58.9%

2.63%

62%

CD38^hi^

CD38^+^

CD86

CD38

0.42%

CD38^hi^

0.61%

2.87%

CD25 FMO

0%

CD19

CD38^+^

CD38

CD86 FMO

CD38 FMO

0.87%

1.31%

PD-L1

PD-L2

PD-L1

0.58%

0%

PD-L1 FMO

PD-L2 FMO

**Fig S2. Flow cytometry gating strategy for characterization of CD24^hi^CD27^+^ B-cells in the blood.** CD24^hi^CD27^+^ Breg cells were analysed according to their expression of CD86, PD-L1, PD-L2, CD38 and CD25. FMO; fluorochrome minus one.

## Fig S3

**A**


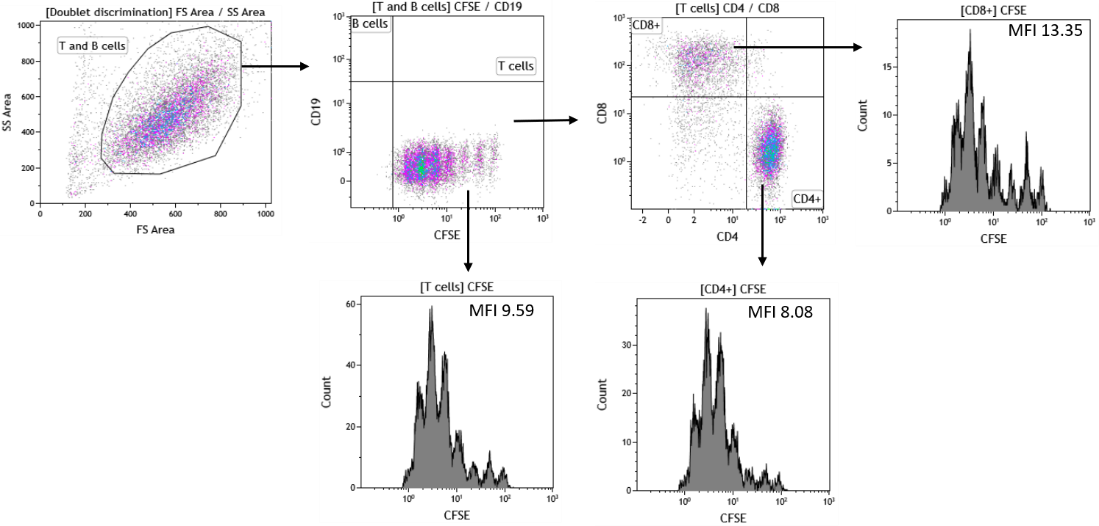


**CD4^+^**

**CD8^+^**

**Total**

**T cells**

**T cells alone**

PI: 2.35

DI: 1.67

PI: 2.86

DI: 2.27

PI: 3.15

DI: 2.74


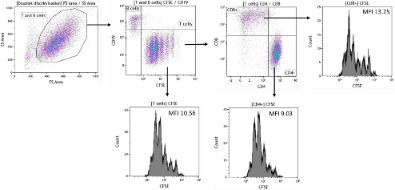


**CD4^+^**

**CD8^+^**

**Total**

**T cells**

**T cells + B cells**

**B2222**

PI: 2.24

DI: 1.89

PI: 2.94

DI: 2.79

PI: 2.64

DI: 2.39

**C**


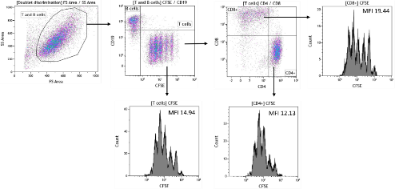


**T cells + B cells**

**+ CpG-B**

**CD4^+^**

**CD8^+^**

**Total**

**T cells**

PI: 2.29

DI: 2.04

PI: 1.96

DI: 1.67

PI: 2.59

DI: 2.43

**Fig S3.** **Flow cytometry gating strategy to evaluate T-cell proliferation in co-culture assay.**

Representative flow cytometry analysis of CFSE-labelled T-cells stimulated with anti-CD3 and anti-CD28 antibodies and cultured **(A)** alone, **(B)** in the presence of B-cells or **(C)** with B-cells and CpG-B for 96 hours. PI; proliferation index, DI; division index

## Fig S4

**
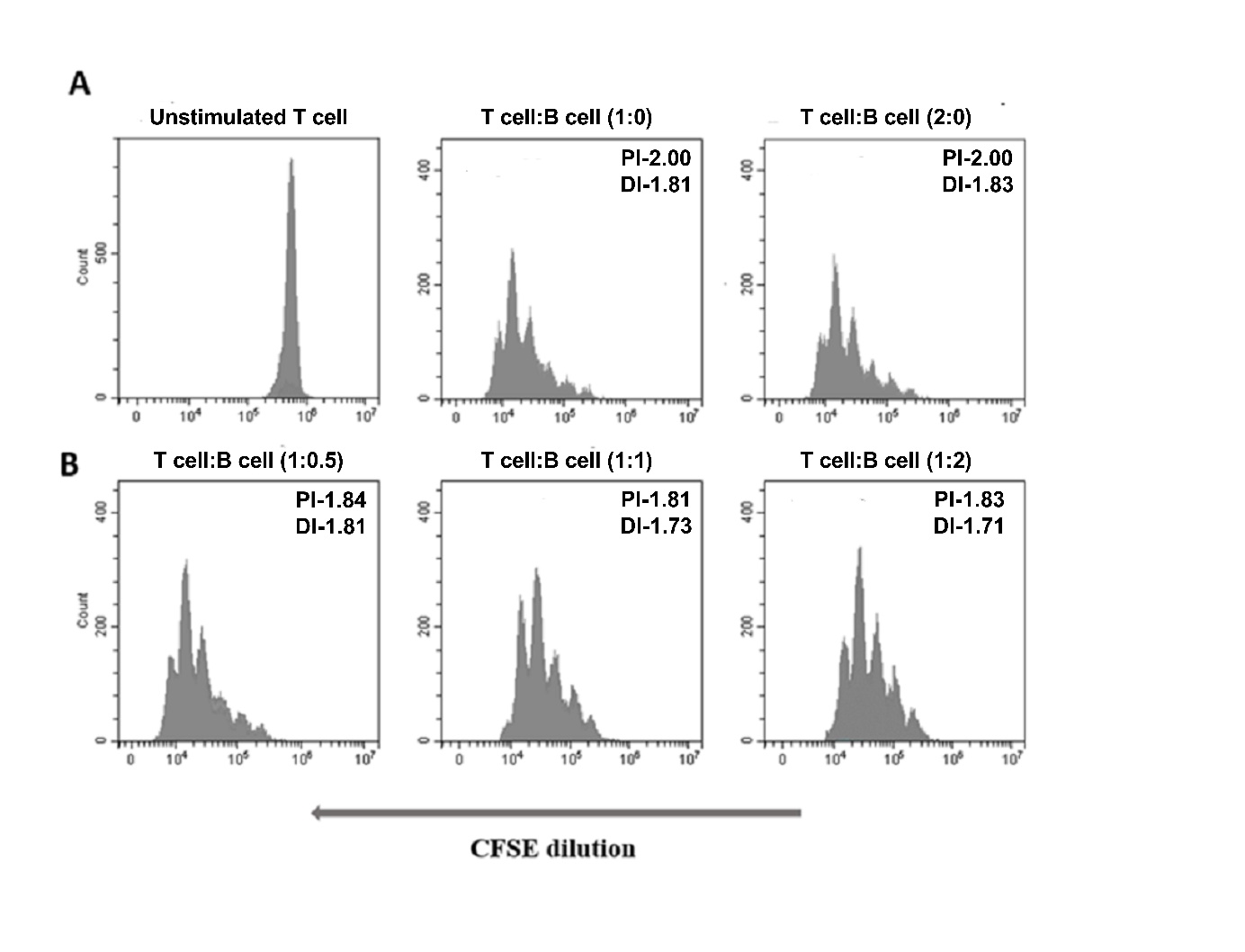
Fig S4. Effect of T-cell density and different T-cell - B-cell ratios on T-cell proliferation.** (A) To evaluate whether T-cell density affected their proliferation we tested two different concentrations: 4x10^4^ (1:0) and 8x10^4^ (2:0) T-cells. T-cell proliferation were similar in both setups. (B) Titration experiments were carried out with different ratios of T-cells and B-cells. In this example, T-cells were cultured with unstimulated B-cells.

**Data sheet**
